# Brain Complete Response to Cabozantinib prior to Radiation Therapy in Metastatic Renal Cell Carcinoma

**DOI:** 10.1155/2019/6769017

**Published:** 2019-02-13

**Authors:** An Uche, Chad Sila, Tad Tanoura, James Yeh, Neil Bhowmick, Edwin Posadas, Robert Figlin, Jun Gong

**Affiliations:** ^1^Division of Hematology/Oncology, Department of Medicine, Harbor-UCLA Medical Center, 1000 W Carson St, Torrance, CA 90509, USA; ^2^Division of Hematology/Oncology, Department of Medicine, Cedars-Sinai Medical Center, 8700 Beverly Blvd, Los Angeles, CA 90048, USA; ^3^Division of Hematology/Oncology, Department of Medicine, Cedars-Sinai Medical Center, 8700 Beverly Blvd, AC1042B, Los Angeles, CA 90048, USA

## Abstract

Cabozantinib represents an established vascular endothelial growth factor- (VEGF-) tyrosine kinase inhibitor (TKI) in the treatment paradigm of metastatic renal cell carcinoma (mRCC). Its activity in mRCC patients with brain metastases (BMs) has been largely underreported in prospective clinical trials. We present the unique case of a heavily pretreated mRCC patient with BMs who achieved a brain complete response to cabozantinib prior to receiving radiation therapy. We end with a literature review and discussion of the biologic rationale and growing evidence supporting the intracranial activity of cabozantinib.

## 1. Introduction

Cabozantinib, a multitarget vascular endothelial growth factor- (VEGF-) tyrosine kinase inhibitor (TKI), is currently approved by the US Food and Drug Administration (FDA) for the first-line treatment of metastatic renal cell carcinoma (mRCC) based on a superior progression-free survival (PFS) benefit over sunitinib in the phase II CABOSUN trial [1] and the second-line and beyond treatment in mRCC based on a superior overall survival (OS), PFS, and overall response rate (ORR) benefit over everolimus in the phase III METEOR trial [2]. Despite the establishment of cabozantinib as a standard of care therapy in the treatment paradigm of mRCC, its activity in mRCC patients with brain metastases (BMs) remains largely unexplored. We present the case of a heavily pretreated mRCC patient with BMs who experienced an unusual brain complete response (CR) to cabozantinib.

## 2. Case Presentation

**Figure 1:**
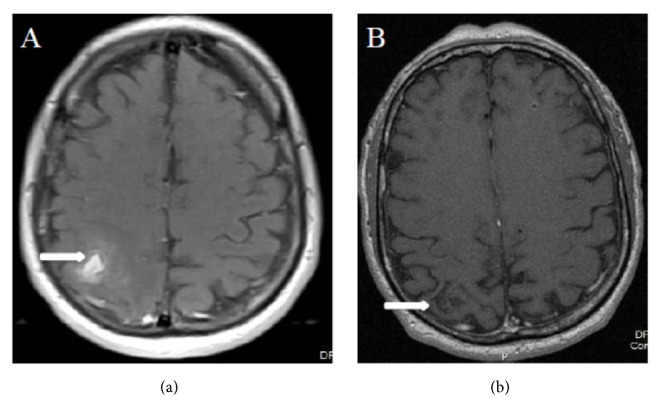
*MRI brain*. (a) An axial, postcontrast-enhanced, fat-saturated T1-weighted image of the brain shows a 2.0 x 1.0 cm right parietal mass with ill-defined area of enhancement. (b) An axial, contrast enhanced, fat-saturated T1-weighted image of the brain, 3 weeks after therapy with cabozantinib, shows resolution of the prior mass and surrounding enhancement.

**Figure 2:**
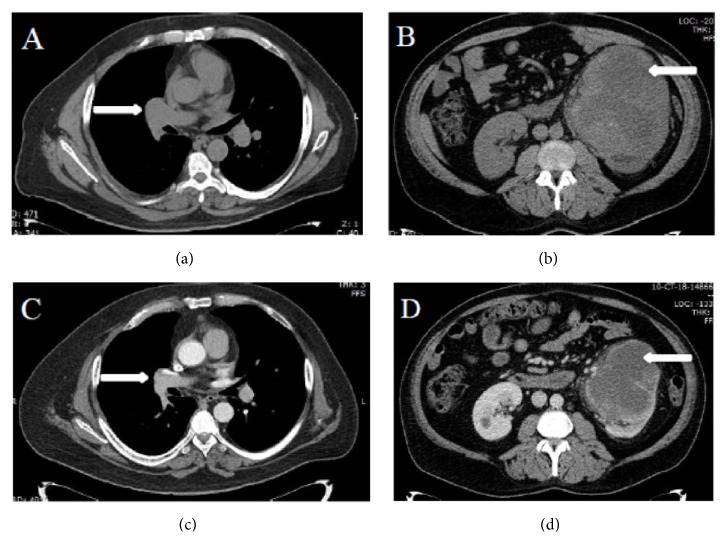
*CT CAP*. (a) and (b) Axial images of CT CAP showed right sided hilar lymphadenopathy and a large left kidney mass. (c) and (d) Axial images of CT CAP showed improvement in right sided hilar lymphadenopathy and reduction in size of left kidney mass, after 8 weeks of therapy with cabozantinib.

The patient is a 48-year-old male who presented with gross hematuria in February 2017. Computed tomography (CT) of the chest, abdomen, and pelvis (CAP) showed bilateral renal masses, numerous bilateral pulmonary nodules, and mediastinal and right hilar lymphadenopathy. Pathology from a transbronchial lymph node biopsy (station 11R) revealed metastatic renal cell carcinoma. He was started on sunitinib 50 mg daily for 14 days every 21 days cycle and experienced a partial response (PR) until April 2018 when he developed worsening flank pain. CT CAP showed progression of disease (PD) with an enlarging right renal mass and right hilar lymphadenopathy. He was started on nivolumab 3 mg/kg every 14 days. After 8 cycles of nivolumab, patient developed worsening headache and blurry vision of the left eye, which prompted a magnetic resonance imaging (MRI) of the brain that showed a 2.5 cm enhancing, right parietal mass associated with hemorrhage and edema as well as punctuate areas of enhancement in the left frontal lobe and left cerebellar peduncle. Of note, a baseline MRI brain obtained after his initial diagnosis was negative for metastatic disease. Repeat CT CAP also showed PD with an enlarging left renal mass and worsening mediastinal lymphadenopathy. Patient was started on third-line cabozantinib 60 mg daily and received a course of dexamethasone 4 mg twice daily with referral to radiation oncology for treatment of his brain metastases. Three weeks after starting cabozantinib, a repeat MRI brain was obtained for radiation planning and showed complete resolution of the right parietal mass with now encephalomalacia of the area (Figure 1). Patient also reported improvement of his headache and blurry vision. Due to resolution of the right parietal mass, radiation therapy was no longer deemed necessary and the patient remains on cabozantinib 60 mg daily. A CT CAP, obtained 8 weeks after initiation of cabozantinib therapy, showed partial response with reduction in size of mediastinal lymphadenopathy and bilateral renal masses (Figure 2).

## 3. Discussion

The median OS for mRCC has improved to approximately 30 months in the current decade from 13 months in the era of cytokine therapy nearly 3 decades ago [3, 4]. Although accounting for approximately 8% of all RCC metastatic sites [5], the survival of mRCC patients with BMs treated in the VEGF-TKI era remains poor with several retrospective series having shown that median OS ranges from 5.4-14.4 months [6–8]. In particular, patients with >4 BMs have a significantly worse OS (3.9 months) compared to those with ≤4 BMs (15.4 months; hazard ratio (HR) 3.02, 95% confidence interval (CI) 1.33-6.83, p=0.005) [6]. VEGF-TKI therapy has been shown to prolong time to BM development (median 28 months) compared to mRCC patients who did not receive TKI therapy (median 11.5 months) though preclinical investigations have demonstrated limited penetration of sorafenib or sunitinb across the blood-brain barrier [9].

Patients with BMs have historically been excluded from most prospective targeted therapy clinical trials in RCC; although the METEOR and CABOSUN trials allowed treatment of mRCC patients having BMs with cabozantinib, this subset was underrepresented (<1%) in METEOR and not reported in CABOSUN [1, 2]. Nevertheless, there exists biologic rationale for efficacy of cabozantinib in mRCC patients with BMs as expression of MET, a target of cabozantinib, was found to be in 35% of BMs compared to 0% of primary RCC tumors [10]. A separate series corroborated findings that cMET expression was significantly higher in metastatic sites compared to primary RCC tumor sites [11]. Furthermore, the ability of cabozantinib to penetrate the central nervous system (CNS) has recently been supported in several tumor types including glioblastoma, RCC metastatic to the brain, and non-small-cell lung cancer metastatic to the brain where cabozantinib demonstrated therapeutic efficacy [12–14].

Data on the efficacy of other commonly used VEGF-TKIs and systemic agents in treating RCC BMs are limited. Expanded access studies of sorafenib have shown a 12-week disease control rate (DCR) 60.7% and a median PFS of 7.4 months, while sunitinib has shown an ORR of 9% (clinical benefit rate or CBR of 42%) and median PFS of 5.3 months in RCC patients with BMs [15, 16]. In an expanded access study of nivolumab, an ORR of 18.8% or CBR of 53% was produced in RCC patients with BMs [17]. Data on the intracranial activity of nivolumab and ipilimumab in RCC BMs is limited, but in a melanoma BM cohort the combination showed an intracranial clinical benefit rate of 57% with a 26% CR rate and 30% PR rate [18]. In a triple-negative breast cancer preclinical study, the mTOR inhibitor temsirolimus had little activity in treating BMs at high concentrations of 100 nM [19]. In mouse models exploring the ability of pazopanib to penetrate the CNS, only 1.5% of the concentration in plasma was able to reach the brain implying severe restriction of this agent for brain penetration [20]. The variable ability of VEGF-TKIs to penetrate the CNS may be dependent on reliance on active uptake through drug transporters. For example, sorafenib and sunitinib showed low-moderate affinity for the ATP-binding cassette (ABC) transporter, ABCB1, where brain penetration was increased 1.9-fold and 2.9-fold for sorafenib and sunitinib, respectively, in knockout mice with the absence of ABCB1 when compared to controls [21].

Our case represents among the initial cases of a brain CR to cabozantinib in a heavily pretreated mRCC patient and adds to a growing body of evidence supporting its intracranial antitumor activity. Notably, nivolumab in combination with ipilimumab and nivolumab monotherapy comprise another FDA-approved standard of care therapy in the first-line and second-line treatment of mRCC, respectively; though patients with BMs were excluded in their registration trials [22, 23]. On this note, cabozantinib may represent a preferable option in mRCC patients with BMs given the current evidence at hand. Further investigation of the intracranial activity of cabozantinib in mRCC is warranted. Results of an ongoing BM-specific clinical trial (NCT02260531) are eagerly awaited in hope that the CNS penetrating ability and pharmacokinetics of cabozantinib will be better understood.

## 4. Conclusion

We have presented an unusual case of a patient with mRCC and BMs experiencing a brain CR to cabozantinib prior to radiation therapy. Our report contributes to the accumulating but overall limited evidence on the intracranial activity of cabozantinib. Further investigation is duly warranted to evaluate the ability of cabozantinib to penetrate the blood-brain barrier and elicit a clinical response. This is especially important in mRCC patients with BMs where this subset has been historically underrepresented in prospective VEGF-TKI clinical trials.
